# Smoking induces shifts in cellular composition and transcriptome within the bronchial mucus barrier

**DOI:** 10.1111/resp.14401

**Published:** 2022-11-22

**Authors:** Senani N. H. Rathnayake, Benedikt Ditz, Jos van Nijnatten, Tayyaba Sadaf, Philip M. Hansbro, Corry A. Brandsma, Wim Timens, Annemarie van Schadewijk, Peter S. Hiemstra, Nick H. T. ten Hacken, Brian Oliver, Huib A. M. Kerstjens, Maarten van den Berge, Alen Faiz

**Affiliations:** ^1^ University of Technology Sydney, Respiratory Bioinformatics and Molecular Biology (RBMB), School of Life Sciences Sydney New South Wales Australia; ^2^ The University of Sydney, Respiratory Cellular and Molecular Biology (RCMB), Woolcock Institute of Medical Research Sydney New South Wales Australia; ^3^ Department of Pulmonary Diseases University of Groningen, University Medical Center Groningen Groningen the Netherlands; ^4^ University of Groningen, University Medical Center Groningen, Groningen Research Institute for Asthma and COPD Groningen the Netherlands; ^5^ Department of Pathology & Medical Biology University of Groningen, University Medical Center Groningen Groningen the Netherlands; ^6^ Centre for Inflammation Centenary Institute, and the University of Technology Sydney, Faculty of Science Sydney New South Wales Australia; ^7^ Department of Pulmonology Leiden University Medical Center Leiden the Netherlands

**Keywords:** bronchial mucus barrier, cellular deconvolution, gene expression, goblet cell, smoking

## Abstract

**Background and Objective:**

Smoking disturbs the bronchial‐mucus‐barrier. This study assesses the cellular composition and gene expression shifts of the bronchial‐mucus‐barrier with smoking to understand the mechanism of mucosal damage by cigarette smoke exposure. We explore whether single‐cell‐RNA‐sequencing (scRNA‐seq) based cellular deconvolution (CD) can predict cell‐type composition in RNA‐seq data.

**Methods:**

RNA‐seq data of bronchial biopsies from three cohorts were analysed using CD. The cohorts included 56 participants with chronic obstructive pulmonary disease [COPD] (38 smokers; 18 ex‐smokers), 77 participants without COPD (40 never‐smokers; 37 smokers) and 16 participants who stopped smoking for 1 year (11 COPD and 5 non‐COPD‐smokers). Differential gene expression was used to investigate gene expression shifts. The CD‐derived goblet cell ratios were validated by correlating with staining‐derived goblet cell ratios from the COPD cohort. Statistics were done in the R software (false discovery rate *p*‐value < 0.05).

**Results:**

Both CD methods indicate a shift in bronchial‐mucus‐barrier cell composition towards goblet cells in COPD and non‐COPD‐smokers compared to ex‐ and never‐smokers. It shows that the effect was reversible within a year of smoking cessation. A reduction of ciliated and basal cells was observed with current smoking, which resolved following smoking cessation. The expression of mucin and sodium channel (ENaC) genes, but not chloride channel genes, were altered in COPD and current smokers compared to never smokers or ex‐smokers. The goblet cell‐derived staining scores correlate with CD‐derived goblet cell ratios.

**Conclusion:**

Smoking alters bronchial‐mucus‐barrier cell composition, transcriptome and increases mucus production. This effect is partly reversible within a year of smoking cessation. CD methodology can predict goblet‐cell percentages from RNA‐seq.

## Abstract 2

We assessed the cellular composition and gene expression shifts of the bronchial‐mucus‐barrier with single‐cell‐RNA‐sequencing‐based cellular deconvolution to predict cell‐type composition in RNA‐seq data. Smoking increased goblet cell abundance in the bronchial‐mucus‐barrier, which is partly reversible after 1‐year smoking cessation. Cellular deconvolution can predict goblet‐cell ratios from RNA‐seq data.

## INTRODUCTION

The mucosal barrier in human airways plays a crucial role in maintaining respiratory health. It acts as the first line of defence against inhaled pathogens and environmental challenges.^1^ This barrier function becomes perturbed in various lung diseases, like chronic obstructive pulmonary disease (COPD). Here, mucociliary clearance is disturbed by defects in ciliary beating, mucin secretion and/or mucus hydration.^2^

Smoking is a major environmental risk factor for perturbing the airway mucosal barrier and developing chronic mucus hypersecretion (CMH) in patients with and without COPD.^3^ However, knowledge regarding underlying mechanisms is incomplete. Laboratory studies indicate that smoke exposure reduces airway surface hydration and increases mucus production, which is predicted to increase total mucin concentration.^4^, ^5^ COPD patients with enhanced sputum mucus viscosity exhibit higher rates of respiratory exacerbations, which might be caused by altered microbiome dynamics.^6^, ^7^ Interestingly, it has been shown that smoking cessation in mild COPD patients and non‐COPD controls is associated with reduced respiratory symptoms and less bronchial epithelial remodelling.^8^, ^9^ This provides strong evidence that smoke‐induced airway mucus biology changes might be partially reversible.

Unbiased transcriptomic sequencing techniques, such as RNA‐seq, enable the investigation of the influence of smoking on the expression of genes involved in regulating the airway mucus barrier. Thus, the expression of gel‐forming and membrane‐tethered mucin and as well as mucous production/secretion‐related transcription factors (MPSTF), such as *SPDEF*, *FOXJ1* and *FOXA2*, can be studied simultaneously.^7^, ^8^ Furthermore, genes involved in airway surface hydration are of interest since mucus hydration plays a central role in mucus viscosity and mucociliary clearance.^2^, ^9^, ^10^ Sufficient airway surface hydration requires a balanced epithelial sodium absorption and secretion of chloride and bicarbonate anions.^2^ Therefore, the expression of airway epithelial sodium channels (ENaC) (*SCNN1A*, *SCNN1B*, *SCNN1D*, *SCNN1G*) and chloride channels (*CFTR*, *ANO1*) is of particular interest.^11^

The cellular deconvolution (CD) concept is a novel way of investigating cell composition in bulk RNA‐seq samples. It provides the ability to assess ratios of cells in already collected bulk RNA‐seq data using scRNA‐seq derived signatures.^12^ CD can thus be an accurate, less time‐consuming and inexpensive way to look at the cellular composition compared to scRNA‐seq.

The ability to investigate the cellular composition changes in human airway samples and cellular level gene expression changes can be a powerful approach to better understand how current smoking alters the cell types and the molecular biology of the airway mucus barrier at the transcriptional level. This study aimed to assess the effects of smoking on cellular composition and transcriptomic differences of genes involved in mucus barrier function in the airway wall of patients with COPD and non‐COPD controls. In addition, we aimed to validate two commonly used CD methods, CIBERSORT and non‐negative least squares (NNLS), using histological staining.

## METHODS

### Patient characterization

**FIGURE 1:**
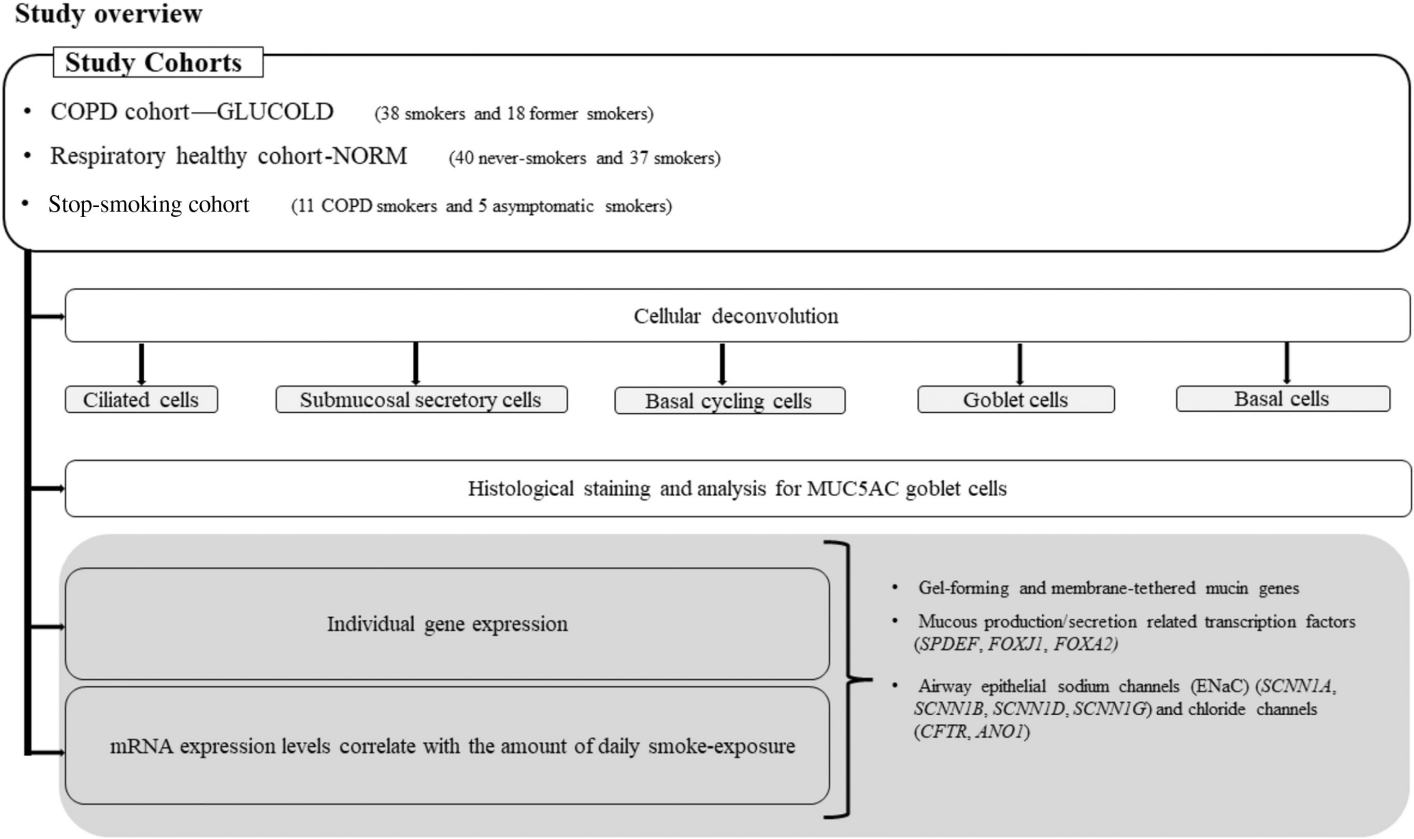
Flow chart of the study procedure.

Bulk RNA‐seq expression was assessed in bronchial biopsies that were previously collected from three cohorts (Figure 1): the first cohort includes 56 COPD patients from the Groningen Leiden Universities and Corticosteroids in Obstructive Lung Disease study (GLUCOLD cohort, ClinicalTrails.gov NCT00158847), the second cohort includes 77 healthy smokers from the Study to Obtain Normal Values of Inflammatory Variables from Healthy Subjects (NORM cohort, ClinicalTrails.gov NCT00848406), and the third cohort includes 11 COPD patients and five healthy smokers from the Stop smoking Cohort. Methods and patient characteristics have been described and summarized in Appendix S1 in the Supporting Information.^13^, ^14^, ^15^

### Sample collection and processing

Sample collection has been described previously for each cohort.^14^, ^16^, ^17^ Briefly, in the GLUCOLD cohort, samples were obtained by fiberoptic bronchoscopy, which was performed using a standardized protocol.^18^, ^19^ Subjects were asked to refrain from smoking on the day of the bronchoscopy. Bronchial biopsies were randomly taken from (sub) segmental carinae in the lung's right or left lower lobe, whereby one part was immediately snap‐frozen and stored at −80°C for later sequencing analyses. In the NORM cohort, bronchial biopsies were collected from the segmental divisions of the main bronchi and stored at −80°C. In the Stop‐smoking cohort, bronchial biopsies were collected from the subcarinal of the right middle or lower lobe and snap‐frozen and held at −80°C. These biopsies were collected from all participants before and after 12 months of smoking cessation.^14^ Appendix S1 in the Supporting Information describes subsequent sample processing, RNA sequencing, quality control and mapping of all three human cohorts.

### Single‐cell RNA sequencing (scRNA‐seq) signatures

scRNA‐seq signatures (for ciliated, goblet, submucosal secretory, basal and basal cyclic cells) were selected, representing the unique profiles of each cell type, as previously explained.^12^, ^20^ Bulk deconvolution was then conducted on the previously described three datasets (GLUCOLD, NORM and STOP smoking) using the NNLS method^21^ and CIBERSORT method.^22^ The resulting deconvolution predicted cell ratios were compared within each dataset between smokers and never‐smokers, using the non‐parametric Mann–Whitney *U* test (GLUCOLD and NORM) and non‐parametric Wilcoxon matched‐pairs signed rank test (STOP smoking).

### Statistical analysis

All RNA‐seq expression data from human bronchial biopsies were analysed using the R statistical software version 3.6. Differential expression profiles of 23 genes, including membrane‐tethered and gel‐forming mucin genes, MPSTF and epithelial sodium and chloride channel genes (Figure 1, Table S1 in the Supporting Information), were assessed using the likelihood ratio testing method in the edgeR package (R‐package version 3.26.6).^23^ A full description of the gene expression analysis can be found in Appendix S1 in the Supporting Information.

## RESULTS

### Participant characteristics

Table 1 summarizes the clinical characteristics of subjects included in the GLUCOLD, NORM and Stop‐smoking studies who had biopsies with RNA of sufficient quality for analysis.

**TABLE 1:** Characteristics of participants in GLUCOLD, NORM and Stop‐smoking cohorts

	GLUCOLD cohort		NORM cohort		Stop‐smoking cohort			
					Before smoke cessation		After 12 months of smoke cessation	
	COPD smokers (*n* = 38)	COPD ex‐smokers (*n* = 18)	Never‐smokers (*n* = 40)	Smoker (*n* = 37)	Healthy subjects (*n* = 5)	COPD‐subjects (*n* = 11)	Healthy subjects (*n* = 5)	COPD‐subjects (*n* = 11)
Age (years)	59.3 ± 7.6	63.3 ± 7.4	38.51 ± 18.86	41.55 ± 15.18	49.2 ± 5	56.2 ± 6.3	49.2 ± 5	56.2 ± 6.3
Sex, (M/F, *n*)	33/5	17/1	20/20	22/15	3/2	6/5	3/2	6/5
BMI (kg/m^2^)	25.0 (22.8–27.8)	26.9 (22.1–28.0)	23.4 ± 3.95	24 ± 3.47	NA	NA	NA	NA
Smoking (pack‐years)	41.8 (34.2–55.6)	39.5. (31.3–52.5)	0	18.75 ± 15.95	23.68 ± 5.99	36.23 ± 14.66	23.68 ± 5.99	36.23 ± 14.66
Post‐BD FEV1 (% predicted)	63.1 (56.4–69.4	65.0 (56.8–70.1)	104.82 ± 10.67	102.8 ± 9.89	107.6 ± 4.93	70.55 ± 18.15	108.66 ± 10.96	73.56 ± 16.86
Post‐BD FEV1/FVC	47.8 (39.9–54.7)	46.8 (40.2–51.1)	83.8 ± 6.75	81.1 ± 5.85	81.29 ± 5.57	46.46 ± 32.09	82.41 + 3.70	55.37 + 23.53

*Note*: Data are presented as mean ± SD or median (interquartile range: 25th–75th percentile).

Abbreviations: FEV1, forced expiratory volume in 1 second; FEV1/VC, forced expiratory volume in 1 second (FEV1) to vital capacity (VC) ratio; FVC, forced vital capacity.

### CD predicted goblet cell ratios are increased in COPD and healthy compared to never‐smokers

**FIGURE 2:**
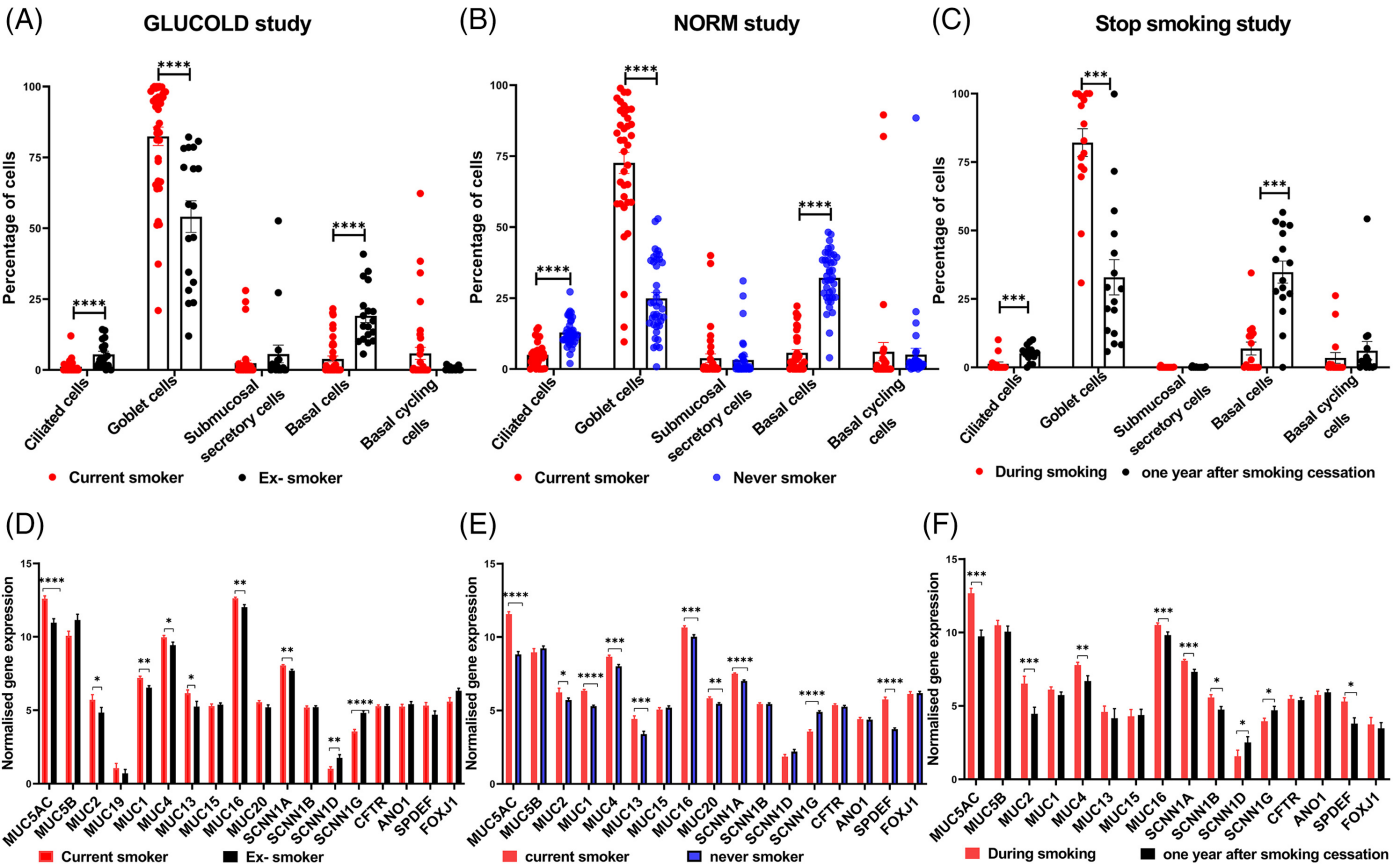
Single‐cell RNA‐seq based cellular deconvolution ratios and gene expression alterations in GLUCOLD, NORM and Stop‐smoking cohorts. Percentage deconvolution of ciliated, goblet, submucosal secretory basal and basal cyclic cells in (A) GLUCOLD cohort, (B) NORM cohort and (C) Stop‐smoking cohort. Differentially expressed mucin and epithelial channel genes in (D) GLUCOLD, (E) NORM and (F) Stop‐smoking cohorts. In GLUCOLD study plots (A) and (D), red and black represent current and ex‐smokers. In NORM cohort plots (B) and (E), red and blue represent current and never‐smokers. In Stop‐smoking cohort plots (C) and (F), samples were clustered based on the visit where red and black dots represent during smoking and 1 year after smoking cessation, respectively. A non‐parametric Mann–Whitney *U* test has been used for all the plots except stop smoking study plots, where a non‐parametric Wilcoxon matched‐pairs signed rank test was conducted. (C) and (F). (**** < 0.0001, *** < 0.002, ** < 0.01 and * < 0.05).

We conducted CD to determine whether there are shifts in airway composition in current‐ versus ex‐ or never‐smokers with available bulk RNA‐seq data in bronchial biopsies. The percentage of goblet cells was higher in COPD and healthy smokers than ex‐smokers and never smokers (*p* < 0.0001). In contrast, ciliated and basal cells were lower in COPD and healthy smokers than ex and never‐smokers (Figure 2A–C). After 1‐year smoking cessation, the percentage of goblet cells reduced (*p* < 0.0002). In contrast, the predicted ratios of basal and ciliated cells increased after 1‐year smoking cessation (*p* < 0.0006), while lower levels were observed in smokers compared to ex‐ and never‐smokers (*p* < 0.0001).

### Transcriptomic level alterations of mucin, MPSTF and ENaC genes by smoking status

In total, 23 mucins, MPSTF, ENaC and chloride channel genes were investigated for differential expression in bronchial biopsies (Table S1 in the Supporting Information). Seven genes were differentially expressed between smokers and ex‐smokers with COPD (false discovery rate [FDR] < 0.05, Figure 2D, Table S2 in the Supporting Information). mRNA expression of *MUC5AC*, *MUC1*, *MUC16*, *MUC2* (mucin‐producing genes) and *SCNN1A* (ENaC‐alpha subunit) was higher in COPD smokers compared to ex‐smokers. In contrast, the expression of *SCNN1G* (ENaC‐gamma subunit) and *SCNN1D* (ENaC‐delta subunit) were lower in COPD smokers compared to ex‐smokers. Further, we performed differential gene expression analysis between healthy current smokers and never‐smokers, where 10 genes were differentially expressed. mRNA expression of *SPDEF*, *MUC5AC*, *MUC1*, *MUC20*, *MUC4*, *MUC13*, *MUC2, MUC16* and *SCNN1A* was higher, whereas expression of *SCNN1G* was lower in COPD and current smokers compared to ex and never‐smokers (Figure 2E, Table S3 in the Supporting Information).

### Expression levels of smoke‐induced mucin, MPSTF and ENaC genes revert after 1‐year of smoking cessation

To evaluate the influence of smoking cessation on the transcriptomics of identified smoke‐induced genes, we analysed bronchial biopsies of 16 smokers with and without COPD, comparing gene expression profiles before and after smoking cessation. After 12 months of smoking cessation, mRNA expression of four mucin‐producing genes was downregulated, including *MUC5AC*, *MUC16*, *MUC2* and *MUC4* (FDR <0.05, Figure 2F, Table S4 in the Supporting Information). *SCNN1B*, *SCNN1A* and *SPDEF* mRNA expression were downregulated (FDR <0.05).

Next, we determined whether mucin, MPSTF, ENaC and chloride channel gene expression was associated with the amount of daily smoke exposure in COPD and healthy current smokers by comparing mRNA expression profiles with the number of cigarettes per day. We focused on smoke‐induced genes differentially expressed in our analysis (Tables S2 and S3 in the Supporting Information) and found that *MUC4* and *SCNN1A* mRNA expression shows no correlation with the number of cigarettes per day in smokers with COPD (FDR > 0.05; Table 2). In healthy smokers, mRNA expression of *MUC5AC* (rho = 0.53, *p* = 0.00079, FDR = 0.008) correlated positively with cigarettes per day, while *SCNN1G* mRNA expression inversely correlated (rho = −0.55, *p* = 0.00046, FDR = 0.008).

**TABLE 2:** Summary of significantly differentially expressed mRNAs related to the amount of daily smoke exposure in smokers with COPD and healthy respiratory smokers

		Genes	Rho	*p*‐value	FDR
Number of cigarettes per day	COPD smokers	MUC4	0.38	0.018	>0.05
		SCNN1A	0.36	0.025	>0.05
	Healthy smokers	MUC5AC	0.53	0.00079	0.008^a^
		MUC16	0.35	0.035	>0.05
		SPDEF	0.38	0.019	>0.05
		SCNCC1G	−0.55	0.00046	0.008^a^

*Note*: This table only shows significant results (*p*‐value < 0.05) before adjusting for multiple testing, applying the Spearman correlation between normalized mRNA expression of genes and amount of daily smoke‐exposure (number of cigarettes per day).

Abbreviations: FDR, false discovery rate; Rho, Spearman's rank correlation coefficient.

^a^ FDR significance: adjusted *p* < 0.05.

### CD derived goblet cell ratios correlate with the MUC5AC staining scores

**FIGURE 3:**
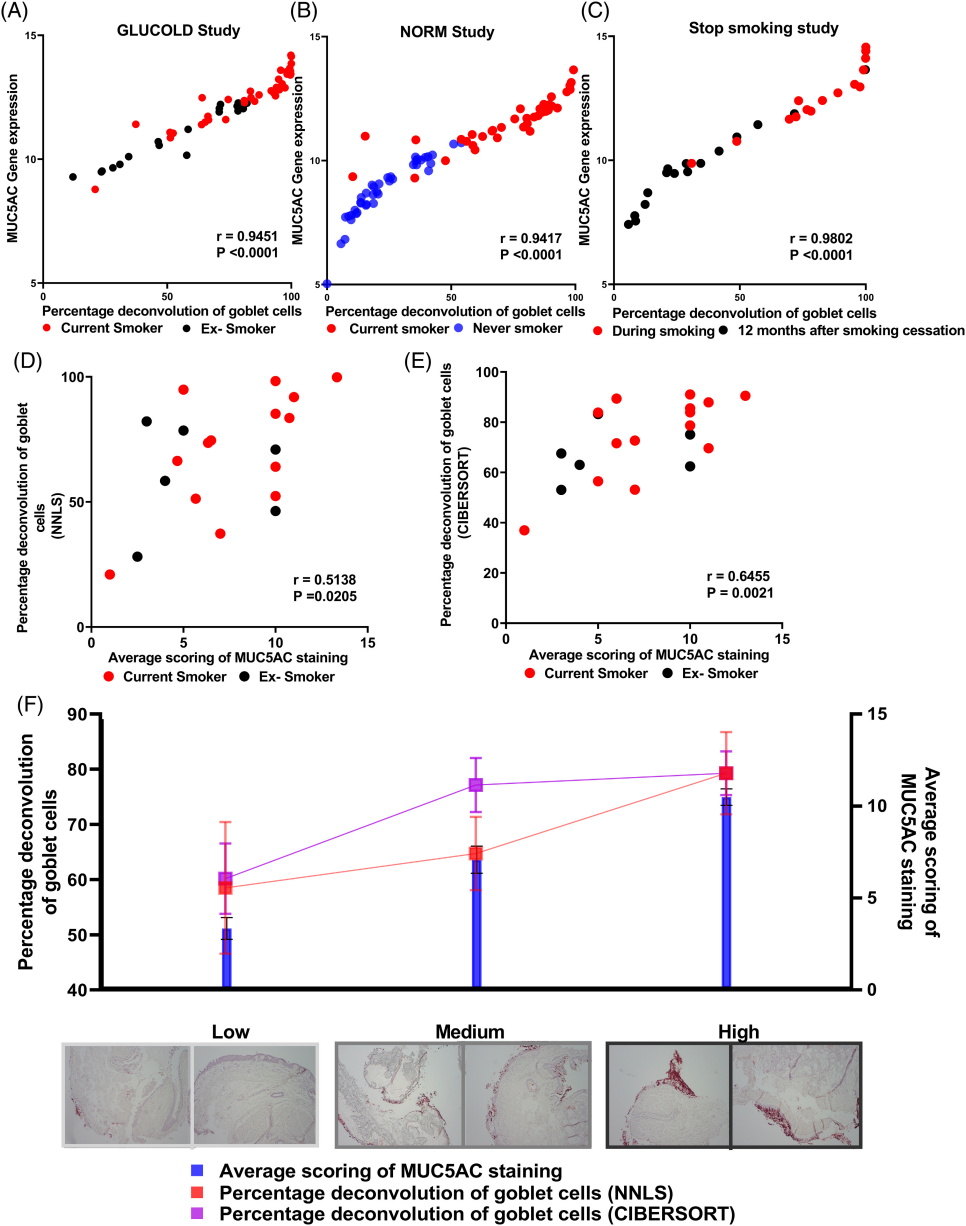
Single‐cell RNA‐seq based percentage goblet cell deconvolution versus MUC5AC gene expression in GLUCOLD, NORM and Stop‐smoking cohorts. (A) MUC5AC gene expression versus percentage deconvolution of goblet cells in GLUCOLD cohort red and black dots represent current and ex‐smokers, respectively. (B) MUC5AC gene expression versus percentage deconvolution of goblet cells in the NORM cohort. Red and blue dots represent current and never‐smokers (C) MUC5AC gene expression versus percentage deconvolution of goblet cells in the stop smoking cohort. Red and black represent the before and 1 year after smoking cessation (D) Percentage deconvolution of goblet cells (NNLS) methods versus average scores of MUC5AC staining in the GLUCOLD cohort. (E) Percentage deconvolution of goblet cells (CIBERSORT) methods versus average scores of MUC5AC staining in GLUCOLD cohort. Red and black dots in the plot (D) and (E) represent current and ex‐smokers, respectively. (F) Representative goblet cell staining from patient samples collected from low mid and high MUC5AC average staining scores‐based groups. Blue bars represent the statistics of average staining scores, while red and purple squares represent percentage goblet cell deconvolution from NNLS and CIBERSORT methods. Plot (A)–(E) Pearson correlation coefficient (*r*) and *p*‐value for each correlation represented.

We performed histological staining to validate the relationship between CD and the actual goblet cell number. Here we associated the CD signature of goblet cells to histological staining (MUC5AC) conducted in adjacent bronchial biopsies taken simultaneously. Initially, we correlated *MUC5AC* gene expression to CD derived goblet cell ratios. This analysis showed a positive correlation in GLUCOLD (*r* = 0.9451, *p* < 0.0001), NORM (*r* = 0.9417, *p* < 0.0001) and Stop‐smoking cohorts (*r* = 0.9802, *p* < 0.0001) (Figure 3A–C). The average scoring of MUC5AC staining in matched bronchial samples of the GLUCOLD study positively correlated with the proportion of goblet cells predicted by CD (NNLS *r* = 0.5138, *p* < 0.0205 and CIBERSORT *r* = 0.6455, *p* < 0.0021; Figure 3D,E). The low, mid and high groups based on the average MUC5AC staining scores show a similar trend with the deconvolution‐based goblet cell percentages (Figure 3F).

**FIGURE 4:**
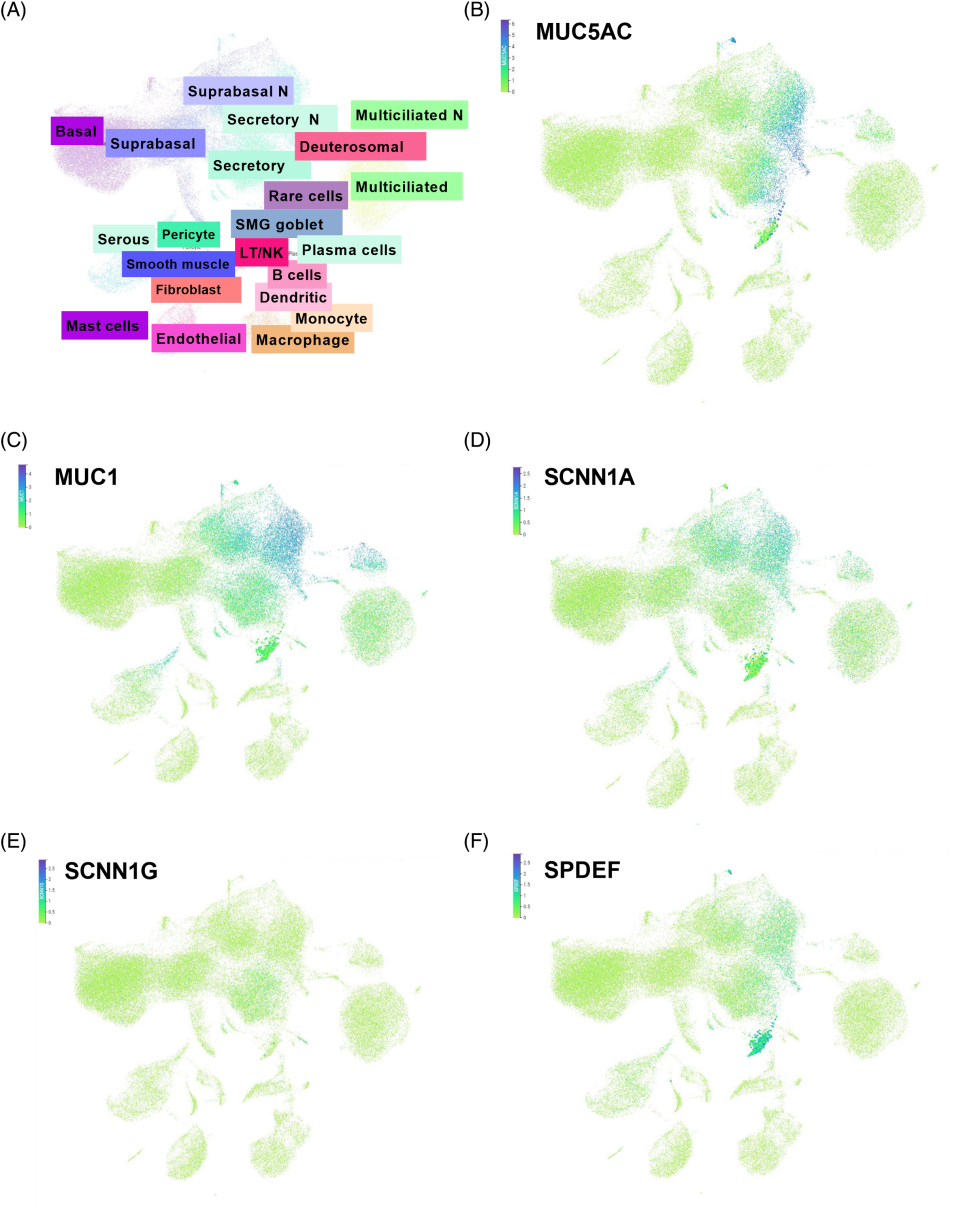
Single‐cell RNA‐sequencing of bronchial biopsies. (A) The overall distribution of cell types. tSNE plots of (B) *MUC5AC*, (C) MUC1, (D) *SCNN1A*, (E) *SCNN1G* and (F) *SPDEF*.

In addition, single‐cell data from bronchial biopsies enabled us to explore which cell types are mainly involved in expressing smoke‐induced mucin‐related and ENaC channel genes. This is of interest since biopsies consist of a mixture of cell types, making it difficult to determine which cells contribute to shifts in gene expression. The gene expression levels of *SPDEF* and mucin (*MUC5AC, MUC1*) were closely correlated to goblet, club and mucociliary cells. In contrast, ENaC genes SCNN1G and SCNN1A were specific to club cells or expressed by all epithelial cell types, respectively **(**Figure 4A–F **).**

## DISCUSSION

**FIGURE 5:**
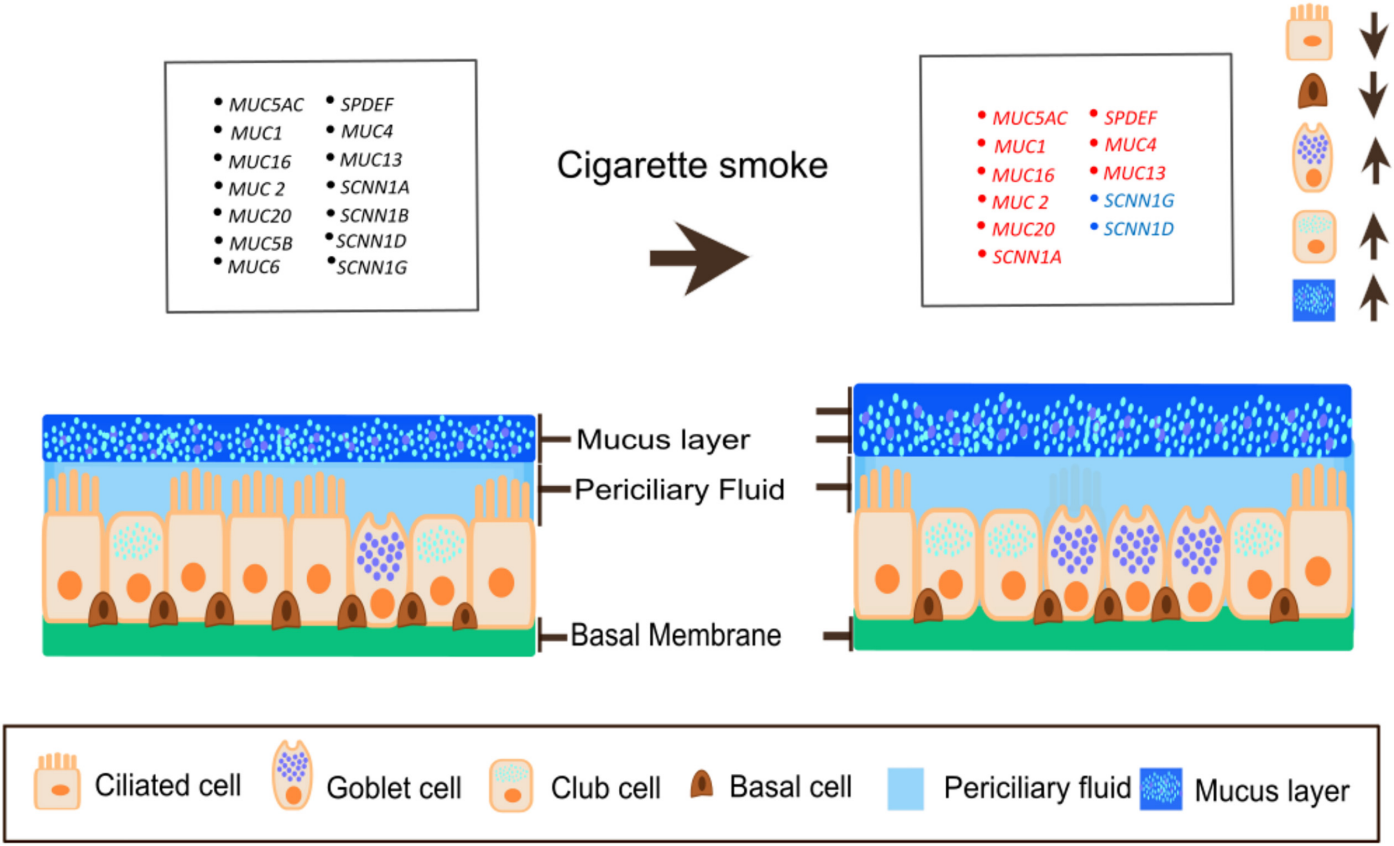
Summary of bronchial epithelial cellular composition shifts upon smoke exposure according to our study findings. The arrows represent decreased ciliated cells and increased‐goblet and club cells with increased mucus layer when smoking compared to never‐smokers. The red and blue colours indicate the increased and decreased expression of the gene with cigarette smoking, respectively.

This study demonstrates that current smoking alters the predicted epithelial cellular compositions and transcriptional profiles of mucins, MPSTF and ENaC genes in COPD and healthy subjects. Whereas the increase in goblet cells in smoking and COPD has been known for some time, our study findings shed more light on which detailed changes happen in the airway mucus barrier, specifically in cell composition and the gene expression changes leading to mucus production; these findings are summarized graphically in Figure 5. Furthermore, we show that both deconvolution techniques that we used in the current study, CIBERSORT and NNLS, provided a good representation of the composition of goblet cells determined by histology.

It has previously been demonstrated that cigarette smoke induces mucin (e.g., *MUC5AC*) gene expression independent of disease status in human airways via growth differentiation factor 15 (GDF15) production, which activates the phosphoinositide 3‐kinase (PI3K) pathway and promotes mucin genes expression.^24^, ^25^, ^26^ Our study mirrors this with *MUC5AC* and other mucin genes, which are more highly expressed in current smokers than ex‐ and never‐smokers, regardless of disease status. Further, our RNA‐seq data of the Stop‐smoking cohort allowed us to assess the impact of smoking cessation, showing that goblet cells decrease along with several mucin genes. Previous studies found that smokers who quit smoking for more than 3.5 years have fewer goblet cells than current smokers and smokers who stopped smoking for less than 3.5 years.^27^ This reversible effect of smoking on goblet cell number aligns with recently reported findings of decreased expression of a subset of mucin genes measured in nasal brushings after smoking cessation.^28^

CD allows us to acquire cell‐specific information from bulk gene expression obtained from heterogeneous tissue samples. This cost‐effective in silico approach allows for the use of bulk gene expression samples to investigate the cellular compositions without immunohistochemical staining or single‐cell sequencing, both a time‐consuming and expensive process that also requires the availability of additional samples.^29^ Our study found a higher proportion of goblet cells and a lower proportion of basal and ciliated cells in current smokers than ex‐ and never‐smokers in COPD patients and healthy smokers. In contrast, these cell populations shifted towards normal after 1 year of smoking cessation. Basal cells play a vital role by acting as a progenitor for secretory and ciliated cells in the airway epithelium to maintain the homeostasis of airway epithelial maintenance and repair.^30^ When analysing the sc‐RNA seq of smokers and never‐smokers' tracheal epithelial cells with trajectory analysis, Goldfarbmuren and colleagues found that hybrid early secretory cells lost their ciliogenic ability and differentiated to ciliated cells upon smoke exposure.^31^ This might explain our findings on the reduced proportion of ciliated cells in COPD and non‐COPD current smokers.

We observed increased expression levels of several membrane‐tethered and gel‐forming mucin genes in current smokers with and without COPD compared to never‐smokers, including *MUC5AC* but not *MUC5B*. Further, our results indicate decreased goblet cell composition, most likely causing the reduced expression of *MUC5AC*.^32^ This is further proved by the significant correlation between *MUC5AC* gene expression, the staining scores generated from histological staining (Figure S1 in the Supporting Information) and when looking at current and never smokers separately as well (Figure S2 in Supporting Information). Additionally, the mRNA expression of *SPDEF*, *MUC20*, *MUC4* and *MUC13* was increased in healthy smokers compared to never smokers. Similar expression directions were observed in COPD smokers.^33^ However, they missed FDR significance, likely due to a greater smoker/never‐smokers ratio.

Airway surface hydration plays a central role in mucous barrier homeostasis, which requires balanced sodium absorption and chloride ions secretion. In vitro results indicate that smoke exposure affects the function of sodium and chloride channels in airway epithelial cells.^34^, ^35^ Our study found that ENaC‐alpha (*SCNN1A*), but not ENaC‐beta (*SCNN1B*) mRNA expression, was increased in COPD and healthy smokers compared to never‐smokers, where their expression decreased after 1‐year smoking cessation. Therefore, it is tempting to speculate that smoking might also lead to hyperactive sodium reabsorption in bronchial airway epithelial cells, which reverses after smoking cessation.

There are several limitations related to our study. Our study focused on assessing the composition and activity of the cells and genes involved in mucus production with airway surface hydration. The CD methods we have used will only provide a relative estimation of the cellular proportions in the given bulk RNA seq sample based on cell‐specific markers.^29^ Still, it will reflect the cell composition providing a window to look at the composition of the cells in bulk RNA samples. The staining method we used for *MUC5AC* gives an average score of cell counts based on the staining image, where it would be subject to patient‐specific variation in each tissue sample. Moreover, the staining would give a score based on the stained area available in the image. Furthermore, bronchial biopsy specimens represent the large airways but not the entire conducting airway. Therefore, our results are related to the large but not peripheral airways. This is important to consider as, for example, the primary site of *MUC5B* expression is found in the distal airways.^26^, ^36^ Also, the cell type composition may differ with the sampling method as sc‐RNA seq can detect the rare cell types, which staining methods failed to distinguish in the tissue samples.^37^ Further research is required on the influence of smoking on *MUC5B* gene expression since *MUC5B* overproduction is associated with COPD severity.^2^, ^36^, ^38^ In addition, the content of submucosal gland tissue in the biopsies is likely to be limited and different among various biopsies. Therefore, our results likely reflect gene activity profiles of surface epithelial cells of the bronchial airways.

In summary, we show that smoke exposure is associated with the cellular composition and transcriptomic differences of the bronchial mucus barrier in COPD and healthy smokers compared to never‐smokers. Some of these alterations are partly reversible after 1 year of smoking cessation. The current analysis has utilized the CD method, which accurately predicts the goblet cell percentage, reflected in the staining scores of MUC5AC. Following the same trend highlights the ability to implement this method to estimate the goblet cell percentages in currently available bulk‐RNA seq samples.

## AUTHOR CONTRIBUTION

**Senani N. H. Rathnayake:** Data curation (lead); formal analysis (lead); investigation (lead); validation (lead); visualization (lead); writing – original draft (lead); writing – review and editing (lead). **Benedikt Ditz:** Data curation (lead); formal analysis (lead); investigation (lead); validation (lead); visualization (equal); writing – original draft (lead); writing – review and editing (equal). **Jos van Nijnatten:** Data curation (equal); formal analysis (equal); writing – original draft (equal); writing – review and editing (equal). **Tayyaba Sadaf:** Data curation (supporting); formal analysis (equal); writing – original draft (equal); writing – review and editing (equal). **Philip M. Hansbro:** Funding acquisition (equal); methodology (equal); project administration (equal); resources (equal); supervision (equal); writing – original draft (equal); writing – review and editing (equal). **Corry A. Brandsma:** Funding acquisition (equal); methodology (equal); project administration (equal); resources (equal); supervision (equal); writing – original draft (equal); writing – review and editing (equal). **Wim Timens:** Funding acquisition (equal); methodology (equal); project administration (equal); resources (equal); supervision (equal); writing – original draft (equal); writing – review and editing (equal). **Annemarie van Schadewijk:** Methodology (supporting); validation (equal); visualization (supporting); writing – review and editing (equal). **Peter S. Hiemstra:** Funding acquisition (equal); methodology (equal); project administration (equal); resources (equal); writing – original draft (equal); writing – review and editing (equal). **Nick H. T. ten Hacken:** Funding acquisition (equal); methodology (equal); project administration (equal); resources (equal); writing – original draft (equal); writing – review and editing (equal). **Brian Oliver:** Funding acquisition (equal); methodology (equal); project administration (equal); resources (equal); supervision (lead); writing – original draft (equal); writing – review and editing (equal). **Huib A. M. Kerstjens:** Funding acquisition (equal); methodology (equal); project administration (equal); resources (equal); supervision (equal); writing – original draft (equal); writing – review and editing (equal). **Maarten van den Berge:** Conceptualization (lead); funding acquisition (equal); methodology (lead); project administration (lead); resources (equal); supervision (lead); validation (equal); writing – original draft (equal); writing – review and editing (equal). **Alen Faiz:** Conceptualization (lead); funding acquisition (equal); methodology (lead); project administration (lead); resources (equal); supervision (lead); validation (equal); writing – original draft (equal); writing – review and editing (equal).

## CONFLICTS OF INTEREST

Dr Kerstjens reports grants from Boehringer Ingelheim, other from Boehringer Ingelheim, grants from GlaxoSmithKline, other from GlaxoSmithKline, grants from Novartis, other from Novartis, outside the submitted work. Dr Timens reports personal fees from Rocche diagnostics/Ventana, personal fees from Merk group Dohme, Personal fees from Bristol‐Myers‐Squibb, and Personal fees from AbbVie outside the submitted work. Dr Hiemstra reports grants from Boehringer Ingelheim, and grants from Galapagos, outside the submitted work. Other authors declared no conflict of interest, financial or otherwise.

## HUMAN ETHICS APPROVAL DECLARATION

The local medical ethics committees approved all three studies, and the following are the ethics approval numbers for each study. GLUCOLD—MEC 98/07/112, NORM—METc2009/007 and Stop‐smoking—MEC 97/007. All subjects gave written informed consent.

## Supporting information


Supporting Information S1

## Data Availability

Data from the NORM (NCT00848406), GLUCOLD (NCT00158847) and stop smoking study cohorts can be accessed through collaboration by contacting Maarten van den Berge.
