# Supplementary material for: Smoking induces shifts in cellular composition and transcriptome within the bronchial mucus barrier
Source: Respirology. 2022 Nov 22;28(2):132–42. doi: 10.1111/resp.14401 (PMC10947540; doi:10.1111/resp.14401)
Supplement: Supplementary file 1 — Supporting Information S1 [file RESP-28-132-s001.docx]

**Supporting Information**

**Smoking induces shifts in cellular composition and transcriptome within the bronchial mucus barrier**

Senani N.H. Rathnayake^1,2#,^ Benedikt Ditz^3,4#,^ Jos van Nijnatten^1,4,6,^ Tayyaba Sadaf^1,5^, Philip M. Hansbro^5^, Corry A. Brandsma^4,6^, Wim Timens^4,6^, Annemarie Schadewijk^7^, Peter S. Hiemstra^7^, Nick H.T. ten Hacken^3,4^, Brian Oliver^2^, Huib A.M. Kerstjens^3,4^, Maarten van den Berge^3,4*^, Alen Faiz^1,2,3*^

#, * contributed equally

**Appendix S1-METHODS**

**Patient characterization**

In the GLUCOLD cohort, all patients had irreversible airflow limitation and at least one of the following symptoms: chronic cough, chronic sputum production, or dyspnea on exertion. All 56 patients were current (N=38) or ex-smokers (≥ 1 month, N=18) with at least ten pack-years of smoking. They did not use a course of oral corticosteroids during the last three months. They had no maintenance treatment with inhaled or oral steroids during the previous six months. All patients were in stable clinical condition. In the NORM cohort, participants were considered asymptomatic controls, defined by an absence of respiratory symptoms, normal lung function with an FEV1/forced vital capacity > 70% and an FEV_1_ >1.2L and no infection of the upper respiratory tract within the last two months before the study and no other disease that could interfere with the results. All participants were smokers with >10 pack years in the Stop Smoking cohort. COPD patients were categorized according to the European Respiratory Society and American Thoracic Society guidelines [1]. All had chronic respiratory symptoms and airway limitations. In contrast, the subjects categorized under the asymptomatic group had no chronic respiratory symptoms or airway obstructions [2].

**Sample processing and sequencing**

For the GLUCOLD study, RNA isolation and processing have been previously described [3]. RNA-seq libraries were sequenced using 50 bp single read mode on the Illumina HiSeq 2500 platform. Samples were depleted off ribosomal RNA using a Ribo-zero Gold Kit. All samples were treated equally, sequenced eight times and eventually put together to diminish batch effects. Quality control (QC) was conducted on the raw sequence data using FastQC version 0.11.5 (<https://github.com/s-andrews/FastQC>). Additionally, a principal component analysis (PCA) was performed to detect outliers. The sequences were then trimmed using the Trimmomatic tool, version 0.33 [4]. Two consecutive computational strategies were applied for transcriptome reconstruction. First, the Spliced Transcripts Alignment to a Reference (STAR) version 2.5.3a was used to align and identify all reads that belong to the human genome [5]. Furthermore, biopsy staining has been described elsewhere [6].

For the NORM study, bronchial biopsies were taken from segmental divisions of the main bronchi and immediately frozen in Tissue-Tek (VWR, Radnor, PA) at -80°C. After thawing at room temperature, biopsies were cut from the blocks when semi-solid. Samples were lysed in 600 μl RLT-plus using an IKA Ultra Turrax T10 Homogenizer. RNA samples were further processed using the TruSeq Stranded Total RNA Sample Preparation Kit (Illumina, San Diego, CA), using an automated procedure in a Caliper Sciclone NGS Workstation (PerkinElmer, Waltham, MA). All cytoplasmic and mitochondria rRNA was removed (RiboZero Gold kit). The obtained cDNA fragment libraries were loaded in pools of multiple samples unto an Illumina HiSeq2500 sequencer using default parameters for paired-end sequencing (2 × 100 bp). The trimmed fastQ files were aligned to build b37 of the human reference genome using HISAT (version 0.1.5), allowing for two mismatches in this procedure [7]. Before gene quantification, SAM tools (version 1.2) was used to sort the aligned reads [8]. The gene-level quantification was performed by HTSeq (version 0.6.1p1) using Ensembl version 75 as gene annotation database [9]. Quality control (QC) metrics were calculated for the raw sequencing data, using the FastQC tool (version 0.11.3). Alignments of 220 subjects were obtained. QC metrics were calculated for the aligned reads using Picard-tools (version 1.130) (URL <http://picard.sourceforge.net>) CollectRnaSeqMetrics, MarkDuplicates, CollectInsertSize-Metrics and SAM tools flag stat. We discarded 36 samples due to poor alignment metrics. In addition, we checked for concordance between sex-linked (XIST and Y-chromosomal genes) gene expression and reported sex. All samples were concordant. This resulted in high-quality RNAseq data from 184 subjects. Subsequently, data from asymptomatic smokers and never-smokers were selected for our analysis.

For the Stop smoking cohort, RNA was extracted using the AllPrep DNA/RNA/miRNA Universal Kit (QIAGEN, Netherlands). RNA-sequencing was performed using Illumina NovaSeq6000 sequencing (Paired-End) on extracted mRNA. Quality control (QC) was conducted on the raw sequence data using FastQC version 0.11.5 (<https://github.com/s-andrews/FastQC>). Additionally, a principal component analysis (PCA) was performed to detect outliers. The sequences were then trimmed using the Trimmomatic tool, version 0.33 [4]. Two consecutive computational strategies were applied for transcriptome reconstruction. First, the Spliced Transcripts Alignment to a Reference (STAR) version 2.5.3a was used to align and identify all reads that belong to the human genome [5].

**MUC5AC goblet cell staining**

From 10 patients with a high MUC5AC gene expression score and 10 with a low score, biopsies were stained for MUC5AC. In short, the biopsies were embedded in paraffin. Following 4uM thick sections were cut and stained for MUC5AC (Thermo Fisher Scientific, 1:300). Envision (HRP.Mouse) (Agilent, Santa Clara) was used for detection, and Vector NovaRed (vector laboratories, Burlingame) served as a chromogen.

Calculating the average scores of MUC5AC staining was done microscopically, evaluating the MUC5AC staining in the areas of the intact epithelium (i.e., more than just basal cells presence). Based on this criterium, between 1 and 4 biopsies per patient were available for analysis. Muc5AC staining was assessed based on Intensity (Ruiter score) and stained area (presence). For every biopsy, the intensity and presence score was multiplied, and per patient, the average of the product of this multiplication was calculated and presented.

**Statistical analysis.** All RNA-Seq expression data from human bronchial biopsies were analyzed using R statistical software version 3.6. Differential expression profiles of 23 genes, including membrane-tethered and gel-forming mucin genes, mucus production/secretion-related transcription factors as well as genes involved in epithelial ion and fluid transport (Supplemental Table S1), were assessed, using the likelihood ratio testing method in the edgeR package (R-package version 3.26.6) [10]. Gene expression profiles were analyzed for the effects of smoking status while correcting for age and gender. Comparisons of gene expression profiles between GLUCOLD and NORM participants were not performed due to technical differences in RNA-sequencing between the two cohorts. Spearman correlation testing was applied to determine the association between normalized gene expression profiles (counts per million, log2 transformed) and smoking behaviour (cigarettes per day, pack-years). In GLUCOLD, information about "cigarettes per day" was not available for one participant excluded from that correlation. To investigate the influence of smoking cessation on gene expression in the Stop smoking cohort, we conducted a paired differential gene expression analysis, regardless of disease status.

**REFERENCES**

1. Celli, B.R., et al., *Standards for the diagnosis and treatment of patients with COPD: a summary of the ATS/ERS position paper.* European Respiratory Journal, 2004. **23**(6): p. 932-946.

2. Willemse, B.W., et al., *Effect of 1-year smoking cessation on airway inflammation in COPD and asymptomatic smokers.* Eur Respir J, 2005. **26**(5): p. 835-45.

3. van den Berge, M., et al., *Airway gene expression in COPD is dynamic with inhaled corticosteroid treatment and reflects biological pathways associated with disease activity.* Thorax, 2014. **69**(1): p. 14-23.

4. Bolger, A.M., M. Lohse, and B. Usadel, *Trimmomatic: a flexible trimmer for Illumina sequence data.* Bioinformatics, 2014. **30**(15): p. 2114-20.

5. Dobin, A., et al., *STAR: ultrafast universal RNA-seq aligner.* Bioinformatics, 2013. **29**(1): p. 15-21.

6. Lapperre, T.S., et al., *Smoking cessation and bronchial epithelial remodelling in COPD: a cross-sectional study.* Respir Res, 2007. **8**: p. 85.

7. Kim, D., B. Langmead, and S.L. Salzberg, *HISAT: a fast spliced aligner with low memory requirements.* Nat Methods, 2015. **12**(4): p. 357-60.

8. Li, H., et al., *The Sequence Alignment/Map format and SAMtools.* Bioinformatics, 2009. **25**(16): p. 2078-9.

9. Anders, S., P.T. Pyl, and W. Huber, *HTSeq--a Python framework to work with high-throughput sequencing data.* Bioinformatics, 2015. **31**(2): p. 166-9.

10. Robinson, M.D., D.J. McCarthy, and G.K. Smyth, *edgeR: a Bioconductor package for differential expression analysis of digital gene expression data.* Bioinformatics, 2010. **26**(1): p. 139-40.

**Table S1. Overview of genes studied concerning airway mucus biology**

| **Gene of interest** | **Description** |
| --- | --- |
| *MUC5AC* | Gel-forming mucin |
| *MUC5B* | Gel-forming mucin |
| *MUC2* | Gel-forming mucin |
| *MUC6* | Gel-forming mucin |
| *MUC19* | Gel-forming mucin |
| *MUC7* | Gel-forming mucin |
| *MUC1* | Membrane-tethered mucin |
| *MUC4* | Membrane-tethered mucin |
| *MUC13* | Membrane-tethered mucin |
| *MUC15* | Membrane-tethered mucin |
| *MUC16* | Membrane-tethered mucin |
| *MUC20* | Membrane-tethered mucin |
| *MUC21* | Membrane-tethered mucin |
| *MUC12* | Membrane-tethered mucin |
| *SPDEF* | SAM pointed domain-containing ETS transcription factor |
| *FOXJ1* | Forkhead box protein J1 |
| *FOXA2* | Forkhead Box A2 |
| *SCNN1A* | Sodium Channel Epithelial 1 Alpha Subunit |
| *SCNN1B* | Sodium Channel Epithelial 1 Beta Subunit |
| *SCNN1D* | Sodium Channel Epithelial 1 Delta Subunit |
| *SCNN1G* | Sodium Channel Epithelial 1 Gamma Subunit |
| *ANO1* | Calcium-activated chloride channel: |
| *CFTR* | CF transmembrane conductance regulator |

**Table S2.** **mRNA expression between smokers versus ex-smokers with COPD (GLUCOLD)**

| Ensymbol ID | logFC | Log CPM | LR | PValue | FDR | Gene name |
| --- | --- | --- | --- | --- | --- | --- |
| ENSG00000166828 | -1.11 | 4.26 | 29.15 | 0.00 | 1.07E-05 | SCNN1G |
| ENSG00000215182 | 1.74 | 12.67 | 27.14 | 0.00 | 2.19E-05 | MUC5AC |
| ENSG00000185499 | 0.76 | 7.22 | 12.70 | 0.00 | 0.004897772 | MUC1 |
| ENSG00000162572 | -0.73 | 1.56 | 10.20 | 0.00 | 0.012537936 | SCNN1D |
| ENSG00000181143 | 0.52 | 12.57 | 9.35 | 0.00 | 0.0174322 | MUC16 |
| ENSG00000111319 | 0.38 | 8.01 | 9.12 | 0.00 | 0.019028933 | SCNN1A |
| ENSG00000198788 | 1.41 | 6.64 | 7.93 | 0.00 | 0.030425844 | MUC2 |
| ENSG00000124664 | 0.86 | 5.65 | 6.56 | 0.01 | 0.052023683 | SPDEF |
| ENSG00000173702 | 0.84 | 6.52 | 5.16 | 0.02 | 0.091912232 | MUC13 |
| ENSG00000145113 | 0.54 | 10.09 | 4.86 | 0.03 | 0.103738977 | MUC4 |
| ENSG00000117983 | -0.96 | 11.76 | 3.66 | 0.06 | 0.167374468 | MUC5B |
| ENSG00000176945 | 0.38 | 5.64 | 3.11 | 0.08 | 0.211089227 | MUC20 |
| ENSG00000205592 | 0.87 | 2.30 | 2.58 | 0.11 | 0.261792074 | MUC19 |
| ENSG00000001626 | 0.11 | 5.36 | 0.57 | 0.45 | 0.65625533 | CFTR |
| ENSG00000169550 | 0.13 | 5.50 | 0.37 | 0.54 | 0.731487291 | MUC15 |
| ENSG00000168447 | 0.06 | 5.30 | 0.17 | 0.68 | 0.830158978 | SCNN1B |
| ENSG00000129654 | -0.13 | 6.34 | 0.15 | 0.70 | 0.840122713 | FOXJ1 |
| ENSG00000131620 | -0.07 | 5.59 | 0.07 | 0.80 | 0.896440204 | ANO1 |

*Abbreviations: ENS ID -Ensymbol Id for the gene, log FC -Log2 fold change, P. Val- P-value, FDR-False Discovery Rate*

**Table S3.** **mRNA expression between healthy smokers versus nonsmokers (NORM)**

| Ensymbol ID | logFC | Log CPM | LR | PValue | FDR | Gene name |
| --- | --- | --- | --- | --- | --- | --- |
| ENSG00000124664 | 2.36 | 5.69 | 166.08 | 0.00 | 7.89E-35 | SPDEF |
| ENSG00000215182 | 2.60 | 12.15 | 132.77 | 0.00 | 8.27E-28 | MUC5AC |
| ENSG00000185499 | 1.22 | 6.49 | 113.78 | 0.00 | 6.24E-24 | MUC1 |
| ENSG00000166828 | -1.11 | 4.87 | 66.25 | 0.00 | 5.82E-14 | SCNN1G |
| ENSG00000111319 | 0.56 | 7.67 | 58.91 | 0.00 | 1.67E-12 | SCNN1A |
| ENSG00000176945 | 0.58 | 5.32 | 26.69 | 0.00 | 5.79E-06 | MUC20 |
| ENSG00000145113 | 0.72 | 8.77 | 21.29 | 0.00 | 6.94E-05 | MUC4 |
| ENSG00000173702 | 1.23 | 4.85 | 21.01 | 0.00 | 7.85E-05 | MUC13 |
| ENSG00000198788 | 1.27 | 7.08 | 20.21 | 0.00 | 0.0001123 | MUC2 |
| ENSG00000181143 | 0.66 | 10.91 | 16.01 | 0.00 | 0.0007188 | MUC16 |
| ENSG00000162572 | -0.29 | 2.60 | 2.67 | 0.10 | 0.2336979 | SCNN1D |
| ENSG00000001626 | 0.16 | 5.64 | 2.31 | 0.13 | 0.2733288 | CFTR |
| ENSG00000117983 | 0.39 | 10.04 | 1.96 | 0.16 | 0.3209422 | MUC5B |
| ENSG00000168447 | 0.08 | 5.81 | 0.64 | 0.42 | 0.6035055 | SCNN1B |
| ENSG00000169550 | -0.10 | 5.94 | 0.38 | 0.54 | 0.7019959 | MUC15 |
| ENSG00000131620 | 0.09 | 5.20 | 0.29 | 0.59 | 0.7403823 | ANO1 |
| ENSG00000129654 | 0.06 | 6.81 | 0.12 | 0.73 | 0.8401573 | FOXJ1 |

*Abbreviations: ENS ID -Ensymbol Id for the gene, log FC -Log2 fold change, P. Val- P-value, FDR-False Discovery Rate*

**Table S4. mRNA expression after smoking cessation in smokers with COPD and asymptomatic smokers (Stop smoking cohort)**

| Ensymbol ID | logFC | Log CPM | LR | PValue | FDR | Gene name |
| --- | --- | --- | --- | --- | --- | --- |
| ENSG00000215182 | -2.91 | 12.51 | 36.23 | 0.00 | 2.39E-06 | MUC5AC |
| ENSG00000198788 | -2.03 | 7.32 | 28.76 | 0.00 | 4.48E-05 | MUC2 |
| ENSG00000111319 | -0.74 | 7.87 | 20.30 | 0.00 | 0.00129063 | SCNN1A |
| ENSG00000145113 | -1.07 | 7.75 | 17.40 | 0.00 | 0.0032486 | MUC4 |
| ENSG00000181143 | -0.69 | 10.42 | 13.13 | 0.00 | 0.01307312 | MUC16 |
| ENSG00000124664 | -1.35 | 5.23 | 11.46 | 0.00 | 0.02199264 | SPDEF |
| ENSG00000168447 | -0.82 | 5.49 | 10.08 | 0.00 | 0.03553319 | SCNN1B |
| ENSG00000166828 | 0.73 | 4.65 | 6.39 | 0.01 | 0.11849343 | SCNN1G |
| ENSG00000162572 | 0.82 | 2.68 | 3.47 | 0.06 | 0.28914661 | SCNN1D |
| ENSG00000117983 | -0.45 | 10.99 | 2.54 | 0.11 | 0.38534306 | MUC5B |
| ENSG00000185499 | -0.37 | 6.15 | 2.44 | 0.12 | 0.39756974 | MUC1 |
| ENSG00000129654 | -0.35 | 4.39 | 0.60 | 0.44 | 0.73155635 | FOXJ1 |
| ENSG00000173702 | -0.37 | 5.34 | 0.56 | 0.45 | 0.74331031 | MUC13 |
| ENSG00000131620 | 0.15 | 6.14 | 0.50 | 0.48 | 0.76001646 | ANO1 |
| ENSG00000001626 | -0.11 | 5.70 | 0.28 | 0.60 | 0.83354146 | CFTR |
| ENSG00000169550 | -0.04 | 4.93 | 0.01 | 0.93 | 0.97661291 | MUC15 |

**Figure S1: The correlation between the average staining scores of goblet cell staining with *MUC5AC* gene expression in the GLUCOLD cohort.**
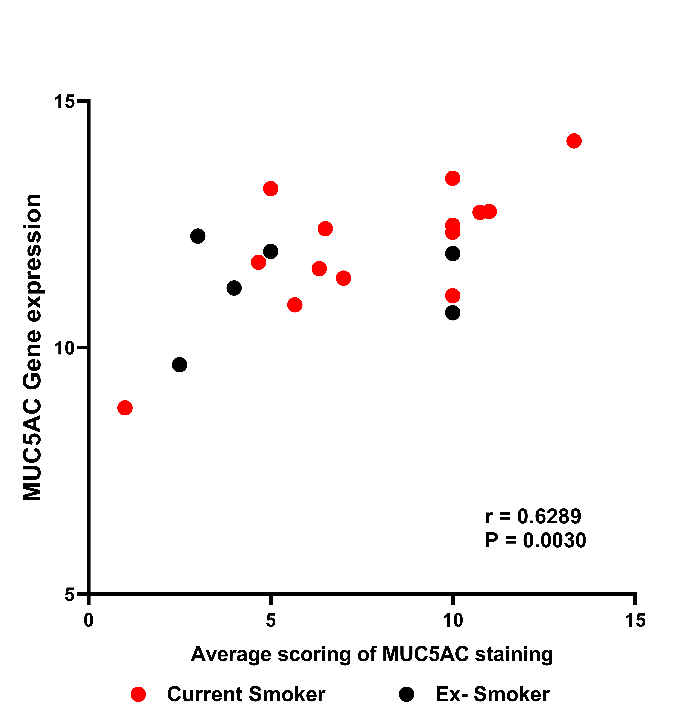


Red and black dots in the plot represent current and ex-smokers, respectively.

*Abbreviations: (r)-* *Pearson correlation coefficient, P- P-value*

**Figure S2:** **Correlation plots of cellular deconvolution derived goblet cell percentages with MUC5AC staining scores of GLUCOLD cohort**
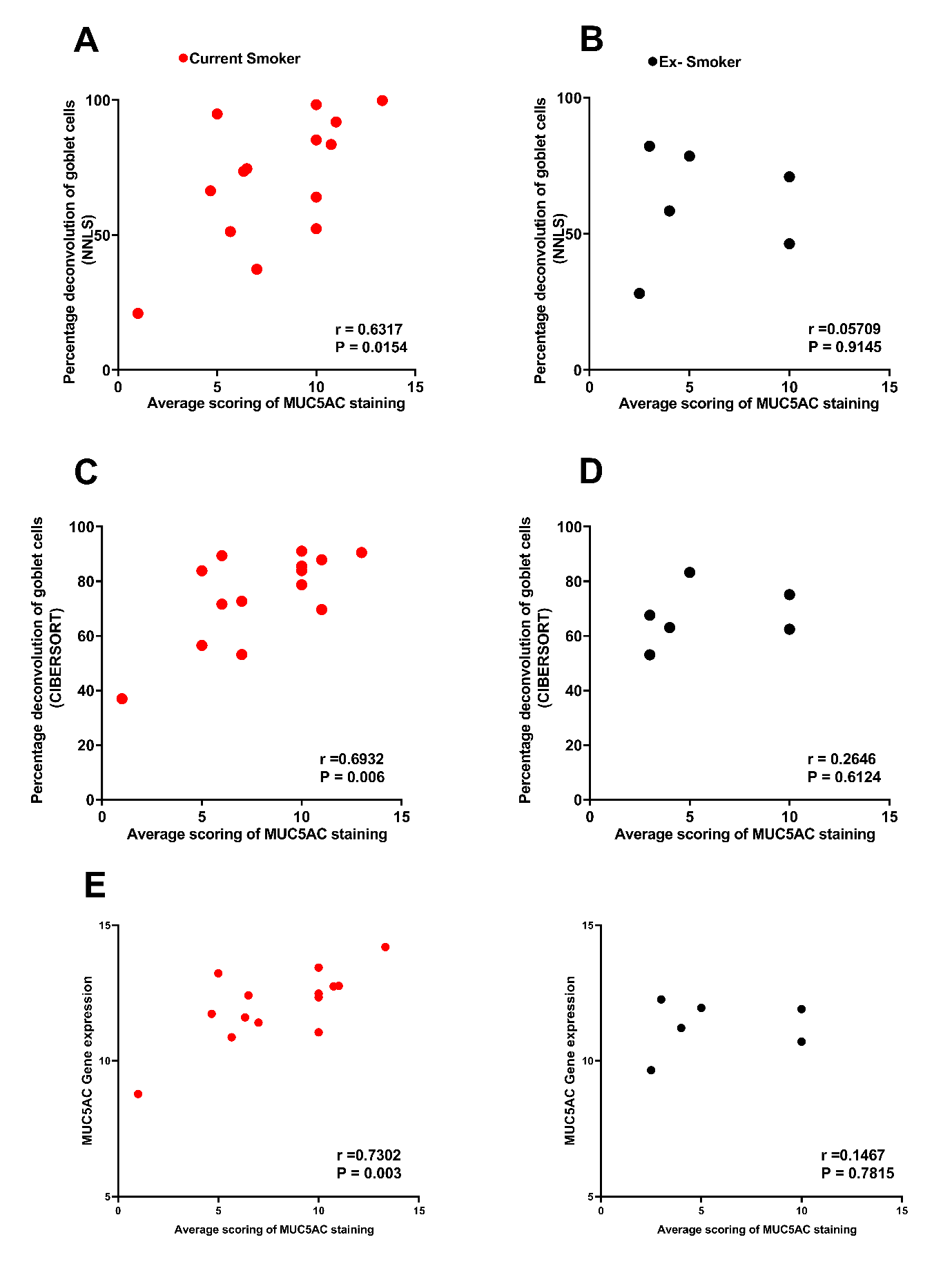


**A)** Percentage deconvolution of goblet cells (NNLS) methods vs average scores of MUC5AC staining in current smoking subjects from the GLUCOLD cohort. **B)** Percentage deconvolution of goblet cells (NNLS) methods vs average scores of MUC5AC staining in ex-smoker subjects from the GLUCOLD cohort. **(C)**  Percentage deconvolution of goblet cells (CIBERSORT) methods vs average scores of MUC5AC staining in current smoking subjects from the GLUCOLD cohort **(D)** Percentage deconvolution of goblet cells (CIBERSORT) methods vs average scores of MUC5AC staining in ex-smoker subjects from the GLUCOLD cohort. **(E)** The correlation between the average staining scores of goblet cell staining with *MUC5AC* gene expression current smoking subjects from the GLUCOLD cohort. **(F)** The correlation between the average staining scores of goblet cell staining with *MUC5AC* gene expression ex-smoker subjects from the GLUCOLD cohort. Plot (A) to (E) Pearson correlation coefficient (r) and p-value for each correlation represented. Red and black dots in the plot represent current and ex-smokers respectively.
